# Genome-Wide Association of Genetic Variants with Intestinal Cholesterol Absorption Markers in a European Population

**DOI:** 10.3390/nu18162679

**Published:** 2026-08-16

**Authors:** Fatma B. A. Mokhtar, Dena A. Nuwaylati, Jogchum Plat, Susan L. M. Coort, Herman E. Popeijus, Marcus E. Kleber, Dieter Lütjohann, Ronald P. Mensink

**Affiliations:** 1 Department of Nutrition and Movement Sciences, NUTRIM Institute of Nutrition and Translational Research in Metabolism, Maastricht University, 6200 MD Maastricht, The Netherlands; fatma.bashir31@gmail.com (F.B.A.M.); dena.nuwaylati@maastrichtuniversity.nl (D.A.N.); j.plat@maastrichtuniversity.nl (J.P.); h.popeijus@maastrichtuniversity.nl (H.E.P.); 2 Division of Clinical Biochemistry, Department of Basic Medical Sciences, College of Medicine, University of Jeddah, Jeddah 23890, Saudi Arabia; 3 Department of Translational Genomics, NUTRIM Institute of Nutrition and Translational Research in Metabolism, Maastricht University, 6200 MD Maastricht, The Netherlands; susan.coort@maastrichtuniversity.nl; 4 Department of Medicine I (Cardiology, Hemostaseology, Medical Intensive Care), Medical Faculty Mannheim, Heidelberg University, 68167 Mannheim, Germany; marcus.kleber@medma.uni-heidelberg.de; 5 SYNLAB MVZ Humangenetik Mannheim, 68163 Mannheim, Germany; 6 Institute of Clinical Chemistry and Clinical Pharmacology, University Hospital Bonn, Venusberg-Campus 1, 53127 Bonn, Germany; dieter.luetjohann@ukbonn.de

**Keywords:** genome-wide association, intestinal cholesterol absorption, plant sterols, single nucleotide polymorphisms

## Abstract

**Background:** Interindividual variability in intestinal cholesterol absorption contributes to differences in serum lipid concentrations and cardiovascular risk. Total cholesterol (TC)-standardized campesterol and sitosterol levels are established markers of cholesterol absorption. However, genetic variants in Europeans associated with these markers remain incompletely characterized. **Methods:** A genome-wide association study (GWAS) was performed in 398 healthy individuals of European ancestry. Samples were genotyped using the Precision Medicine Research Array (PMRA). After quality control, 166,037 common genetic variants with a minor allele frequency (MAF) > 20% were analyzed. Associations between genetic variants and intestinal cholesterol absorption markers (campesterol/TC and sitosterol/TC) were evaluated using additive and recessive genetic models. **Results:** A total of 16 SNPs were identified. Eight SNPs overlapped with both campesterol/TC and sitosterol/TC, of which 2 reached genome-wide significance. Six overlapping SNPs were associated with higher concentrations of both markers: 3 SNPs in *ABCG8* (rs4299376, rs6544713, and rs4245791), 1 SNP in *ADAM12* (rs4962526), and 2 SNPs in non-coding regions (rs260769 and rs5011112). Additionally, two SNPs (rs2033254 and rs12708980) in *CETP* were associated with lower concentrations of these markers. Five of the identified SNPs have not previously been linked to markers of intestinal cholesterol absorption. **Conclusions:** This GWAS confirmed previously reported associations within *ABCG8* and identified candidate loci in *CETP* and *ADAM12* that may be involved in intestinal cholesterol absorption. These findings contribute to our understanding of genetic factors underlying intestinal cholesterol absorption and highlight candidate loci for future replication and functional studies.

## 1. Introduction

Intestinal cholesterol absorption is a multistep process involving several transporters and is determined by the influx and efflux of cholesterol across the enterocyte lining [1]. It is well known that at the apical side, Niemann-Pick C1-Like 1 (NPC1L1) regulates the influx of not only cholesterol but also of plant sterols into enterocytes [2]. At the basolateral side, transintestinal cholesterol excretion (TICE) represents another pathway that delivers cholesterol to the enterocyte [3]. Furthermore, enterocytes synthesize cholesterol themselves, which further fuels the intracellular cholesterol pool [4]. The heterodimer ATP-binding cassette (ABC) transporters G5 and G8 are involved in the apical efflux of cholesterol back into the intestinal lumen, whereas cholesterol can also leave the enterocytes at the basolateral side after being incorporated into chylomicrons [5]. Together, these tightly regulated and interacting processes, along with genetic variability, contribute to the variability in cholesterol absorption among individuals [6].

Individuals can be phenotypically characterized as predominantly cholesterol absorbers or cholesterol synthesizers. In general, people with a high fractional intestinal cholesterol absorption have a relatively low endogenous cholesterol synthesis, and vice versa. These metabolic phenotypes may help to explain interindividual variability in changes in serum low-density lipoprotein cholesterol (LDL-C) concentrations in response to dietary or drug interventions [7]. Dietary plant sterols and stanols (PS), for example, inhibit intestinal cholesterol absorption and consequently lower serum LDL-C concentrations [8]. However, responses may relate to genetic variations [8], as effects of consuming 2 g/day of PS on LDL-C were related to a single nucleotide polymorphism (SNP) in the ATP binding cassette subfamily G member 8 (*ABCG8*) gene [9]. Homozygous carriers of this SNP experienced a greater reduction in serum LDL-C concentrations than heterozygous carriers. Similarly, pharmacogenetic studies suggest that a haplotype in *NPC1L1* is significantly associated with interindividual variation in serum LDL-C responses to ezetimibe treatment [10], a drug that selectively inhibits the NPC1L1 transporter. However, genetic determinants influencing intestinal cholesterol absorption efficiency remain poorly understood, as no genome-wide studies have previously investigated these associations in generally healthy populations. Identifying new SNPs may aid in discovering previously unrecognized genes that are part of the complex intestinal cholesterol absorption network. Improved insight into these mechanisms could ultimately support the development of personalized dietary recommendations and targeted therapies to lower serum LDL-C concentrations. Therefore, the aim of this study was to identify genome-wide associations between SNPs and total cholesterol-standardized (TC-standardized) campesterol and sitosterol concentrations, which are validated markers for intestinal cholesterol absorption [11], in a European population.

## 2. Materials and Methods

### 2.1. Study Population and Biochemical Analyses

The study population was derived from five human intervention studies carried out between 1997 and 2012 at Maastricht University (Maastricht, The Netherlands). The population comprised apparently healthy adults (≥18 years old) of European ancestry. None of the participants used medications known to affect lipid metabolism. Written informed consent was obtained from all participants before the start of the study, and all studies were approved by the Medical Ethical Committee of the University Hospital Maastricht/Maastricht University (METC azM/UM). Details of the studies and biochemical measurements have been previously described [12].

Briefly, campesterol and sitosterol were quantified by gas chromatography with flame-ionization detection (GC-FID) in four of the studies, while GC-mass spectrometry (GC-MS) was used in the fifth study. Both markers are reported as TC-standardized ratios (10^2^ × µmol/mmol TC) to adjust for differences in concentrations of serum lipoproteins that transport campesterol and sitosterol.

### 2.2. DNA Isolation and Genotyping

Genomic DNA samples were available for 475 individuals. DNA was isolated using the QIAamp genomic DNA isolation kit (Westburg BV, Leusden, The Netherlands) according to the manufacturer’s instructions from whole blood or buffy coats. The purity of the DNA was determined by calculating the ratio of the absorbances at 260 and 280 nm (A_260_/A_280_) and at 260 and 230 nm (A_260_/A_230_) (NanoDrop ND-1000 spectrophotometer, Isogen, Lifescience B.V., De Meern, The Netherlands). For all samples, ratios were acceptable and ranged between 1.7 and 1.9 and around 2.0 and 2.2, respectively. DNA concentrations were determined, assuming that an A_260_ of 1.0 corresponds to 50 µg/mL DNA. After thawing, DNA integrity was tested in a random 5% of the samples on agarose gels, which were all deemed adequate for genotyping. Samples were genotyped using the Precision Medicine Research Arrays (PMRA, Thermo Fisher Scientific, Waltham, MA, USA) [13] according to the manufacturer’s instructions. Initial quality control (QC) was performed at the array level. This revealed 2 samples with low analytical quality and 2 wells devoid of DNA; therefore, these 4 samples were excluded. Thus, genotyped samples from 471 individuals were available for further QC.

### 2.3. Genotyping Quality Control

Prior to genome-wide analysis, the genotyped data from 471 individuals with 879,761 SNPs underwent standard QC procedures using PLINK v1.9 software [14]. First, 41 related individuals were excluded as determined by a PI_HAT threshold of >0.2. Next, 1481 SNPs with >2% missing data were removed, followed by the exclusion of 9 individuals with a discrepancy between genotyped sex and clinical records as tested by the F-value (inbreeding coefficient). Moreover, 283 SNPs deviating from Hardy–Weinberg equilibrium (HWE) at a *p*-value of <1 × 10^−10^ were removed (Figure S1). Finally, 11 individuals with a heterozygosity rate ± 3 standard deviations (SDs) from the mean heterozygosity rate of the sample were excluded (Figure S2).

Following these QC steps, principal component analysis (PCA) was applied to assess population structure. For this, linkage disequilibrium (LD) pruning was performed first to remove highly correlated SNPs to generate a set of independent, high-quality SNPs, ensuring that the PCA reflected genome-wide ancestry patterns rather than local LD structure. PCA was then conducted on the LD-pruned set of 233,700 SNPs, and the first 10 principal components (PCs) were extracted (Figures S3 and S4). LD pruning was performed as an intermediary step for PCA and did not permanently remove any SNPs from the dataset.

**Figure 1:**
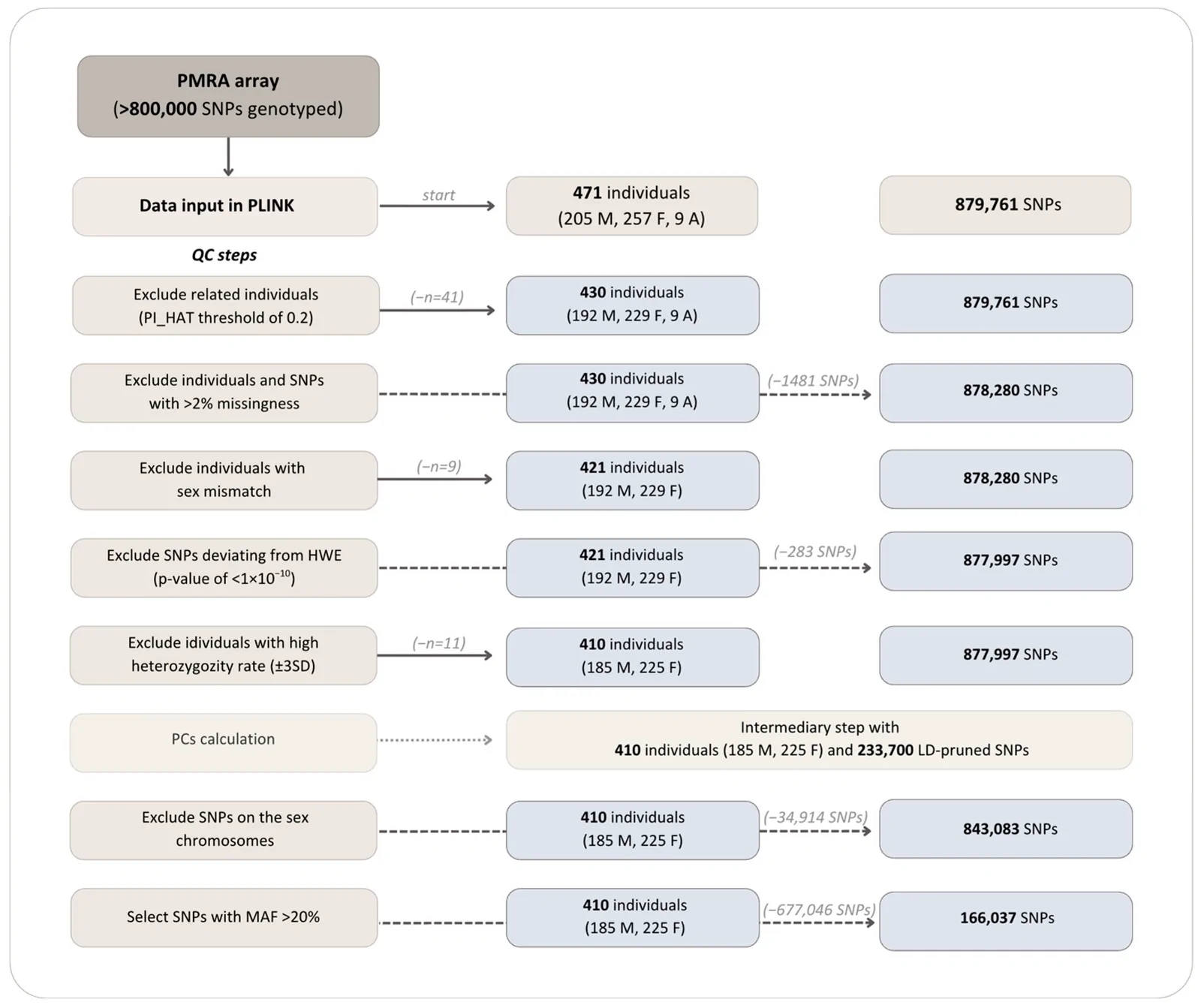
Flow chart of the quality control (QC) steps followed in the study. Abbreviations: PMRA, Precision Medicine Research Array; SNPs, single nucleotide polymorphisms; QC, quality control; PI-_HAT, proportion of identity by descent; M, male; F, female; A, ambiguous, HWE, Hardy–Weinberg equilibrium; PCs, principal components; LD, linkage disequilibrium; MAF, minor allele frequency. Solid arrows indicate individuals exclusions while dashed arrows indicate SNPs exclusions.

Subsequently, 34,914 SNPs located on the sex chromosomes were excluded, and only SNPs with a minor allele frequency (MAF) > 20% were selected (Figure S5). Given the relatively modest sample size, this threshold was applied to reduce low-frequency genotype group sizes, particularly for minor allele carriers, thereby improving the stability and reliability of the association analyses. Finally, 410 individuals and 166,037 SNPs passed the QC criteria. The quality control workflow is summarized in Figure 1.

### 2.4. Statistical Analysis

Baseline characteristics are presented as mean ± standard deviation (SD) for continuous variables and as counts for categorical variables. Differences between the five studies were assessed using analysis of variance (ANOVA) for continuous variables and the chi-square test for categorical variables. Phenotypic data were unavailable for one individual, and BMI data were missing for an additional 11 participants. These individuals were excluded, resulting in a final study population of 398 participants.

Genome-wide association analyses were performed using PLINK v2.0 software [15]. The minor allele within the study population was defined as the effect allele. Linear regression was performed under an additive genetic model, the standard primary approach in GWAS, in which each additional copy of the minor allele was assumed to be associated with a constant change in the continuous phenotype, with the major allele serving as the reference. To investigate potential non-additive genetic effects, recessive models were additionally tabulated as a complementary exploratory analysis for variants with at least 30 minor allele homozygotes, thereby avoiding analyses based on very small minor homozygote groups, as recessive associations are dependent on this genotype group. In the recessive model, minor allele homozygotes were compared with the combined group of heterozygotes and major allele homozygotes. A dominant genetic model was also evaluated, but did not identify additional independent loci beyond those detected under the additive model and was therefore not considered further.

All association analyses were adjusted for sex, body mass index (BMI), ‘Study’ and the first two principal components (PC1 and PC2). Sex and BMI were included because they are established determinants of cholesterol metabolism. To account for residual population stratification, the first 10 PCs were calculated, and PC1 and PC2 were selected based on the observed inflection in the scree plot (Figure S3). ‘Study’ was included as a categorical covariate to account for potential heterogeneity arising from pooling participants from the five studies.

Genome-wide statistical significance was defined as *p* < 5 × 10^−8^, whereas *p* < 1 × 10^−5^ was considered suggestive evidence of an association. Association results were visualized in R v4.5.1 using the ‘qqman’ package v0.1.9 to generate Manhattan plots and quantile-quantile (Q-Q) plots [16]. Genomic inflation was assessed separately for the additive and recessive models using the genomic inflation factor (lambda; λ^GC^) calculated in PLINK to detect potential systematic bias in the association tests and visually through inspection of the Q-Q plots [16].

SNPs reaching genome-wide or suggestive significance for both campesterol/TC and sitosterol/TC were considered stronger candidate loci, as these complementary markers both reflect intestinal cholesterol absorption. Concordant associations across both phenotypes reduced the likelihood of phenotype-specific or chance findings and therefore provide stronger evidence that the identified loci are related to intestinal cholesterol absorption. These loci were subsequently examined for pairwise linkage disequilibrium (LD) within a 500 kb window using Haploview v4.2 [17]. Strong LD was defined by a threshold of r^2^ ≥ 0.8. Haplotype blocks were constructed using the default Haploview algorithm when at least 95% of informative SNPs were in strong LD [18]. Tag SNPs were identified using a likelihood odds ratio (LOD) threshold of 3.0 and a threshold of r^2^ ≥ 0.

## 3. Results

**Figure 2:**
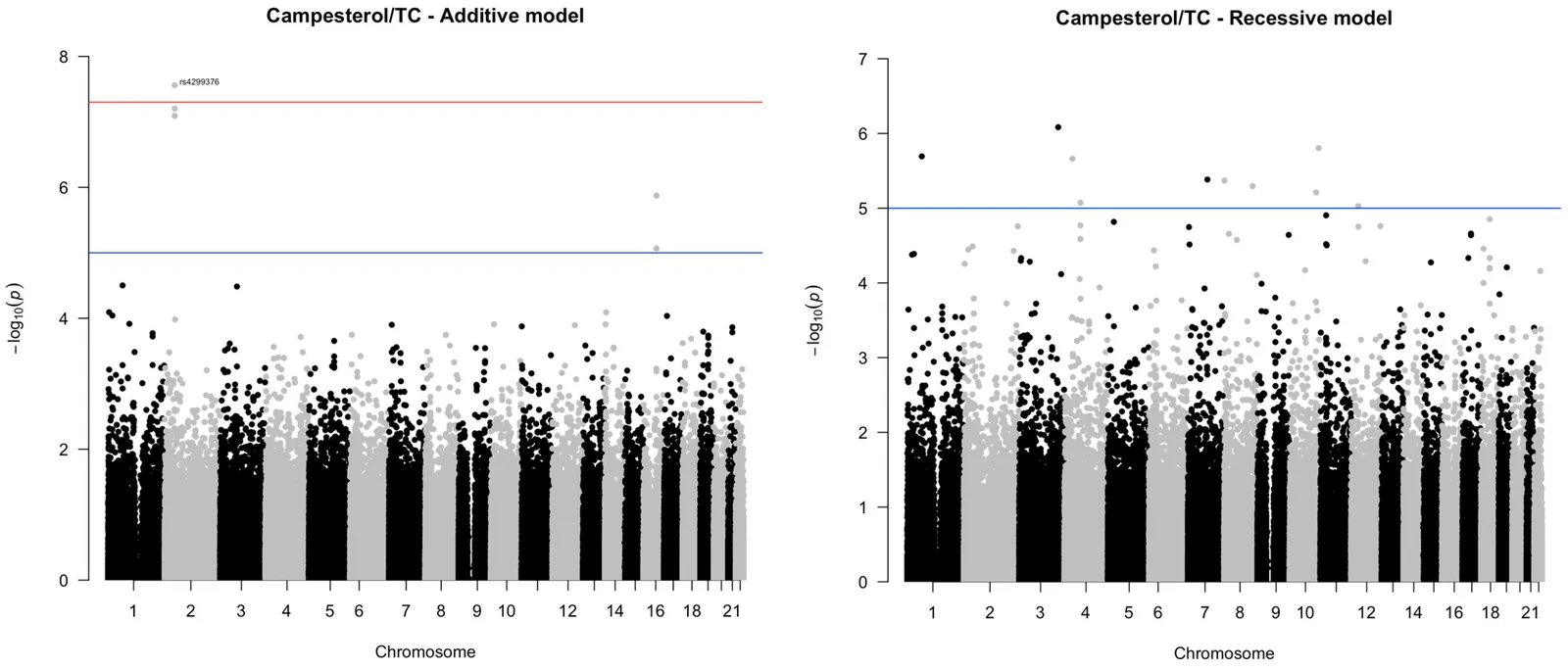
Manhattan plots of association results for serum campesterol/TC levels using the additive (**left**) and recessive (**right**) models. The *y*-axis represents the −log_10_ (*p* values), and the *x*-axis displays variants (dots) according to their chromosomal locations. The horizontal red line represents the threshold for genome-wide significance (*p* = 5 × 10^−8^), while the blue line represents the threshold for suggestive significance (*p* = 1 × 10^−5^).

**Figure 3:**
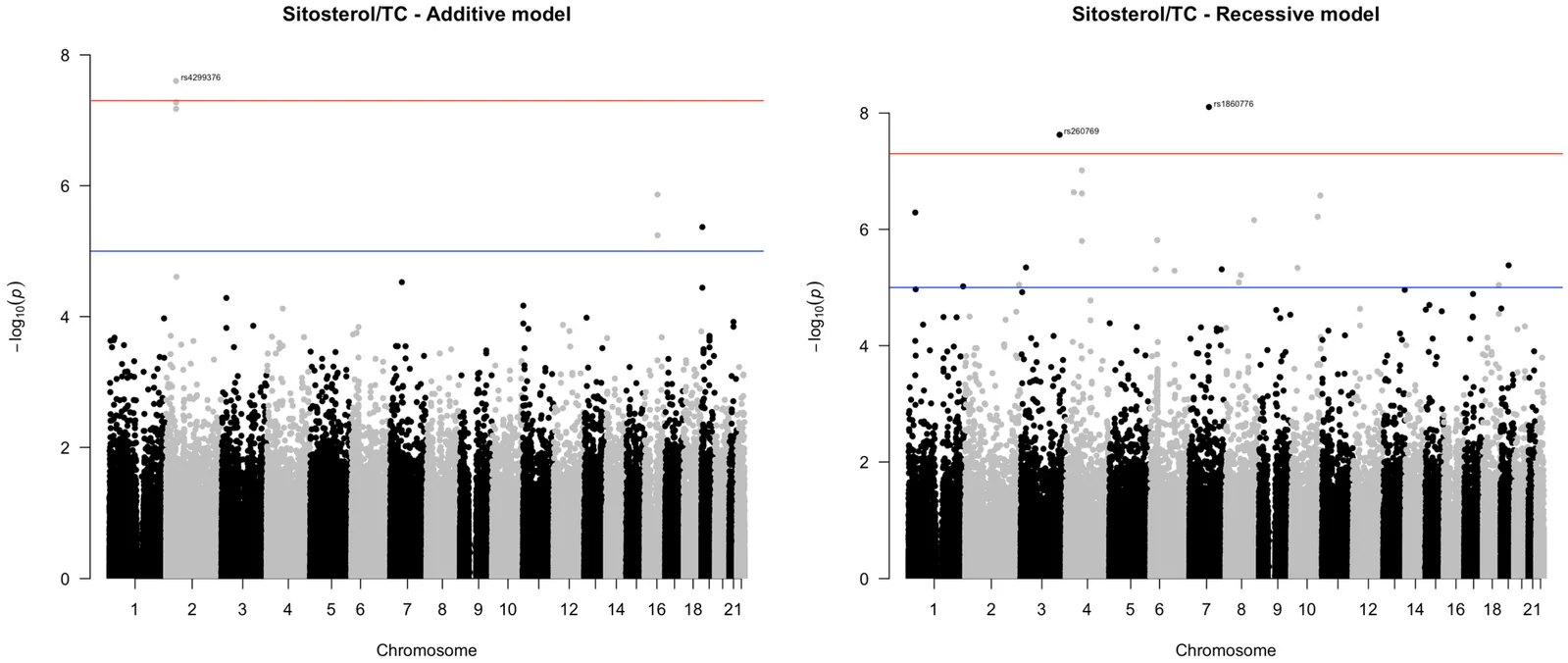
Manhattan plots of association results for serum sitosterol/TC levels using the additive (**left**) and recessive (**right**) models. The *y*-axis represents the −log_10_ (*p* values), and the *x*-axis displays variants (dots) according to their chromosomal locations. The horizontal red line represents the threshold for genome-wide significance (*p* = 5 × 10^−8^), while the blue line represents the threshold for suggestive significance (*p* = 1 × 10^−5^).

The baseline characteristics of the study population are presented in Table S1. Manhattan plots from the genome-wide association analyses under additive and recessive models for both campesterol/TC and sitosterol/TC levels are shown in Figure 2 and Figure 3. No evidence of genomic inflation was observed for any of the tested associations, as indicated by the genomic inflation factor (λ^GC^ = 0.99 for both additive and recessive models for campesterol/TC; λ^GC^ = 1 and 0.99 for additive and recessive models, respectively, for sitosterol/TC) and as observed on the QQ-plots (Figure S6). In total, 16 SNPs were identified using either the additive or recessive models, of which 2 reached genome-wide significance. Table S2 presents TC-standardized campesterol and sitosterol levels according to genotype group for the identified SNPs. Of the 16 SNPs, 8 were associated with both campesterol/TC and sitosterol/TC, while 1 and 7 SNPs were uniquely associated with campesterol/TC or sitosterol/TC, respectively. Table 1 summarizes the identified SNPs, their genomic locations, variants’ consequences, and allele and genotype frequencies within the study population (n = 398).

### 3.1. Associations Overlapping with Both Markers of Intestinal Cholesterol Absorption

Among the 8 SNPs associated with both campesterol/TC and sitosterol/TC, 3 intronic SNPs with additive effects were identified in the ATP binding cassette subfamily G member 8 (*ABCG8*) gene: rs4299376, which reached genome-wide significance; and rs6544713 and rs4245791, which showed suggestive significance for both markers. In addition, two intronic SNPs (rs2033254 and rs12708980) in the cholesteryl ester transfer protein (*CETP*) gene reached suggestive significance under the additive model.

Three additional SNPs were identified using the recessive model. Of these, only rs4962526 was located within an intron of a protein-coding gene (ADAM metallopeptidase domain 12 (*ADAM12*) gene), with suggestive significance for both markers.

Two other SNPs were located in intergenic regions. Of those, rs260769 reached genome-wide significance for sitosterol/TC and suggestive significance for campesterol/TC, whereas rs5011112 showed suggestive significance for both markers. Overall, 6 of the identified SNPs were associated with higher campesterol/TC and sitosterol/TC, whereas the 2 SNPs within the *CETP* gene were associated with lower levels of both markers. The β coefficients of the 8 SNPs for each marker are presented in Table 2. Boxplots of campesterol/TC and sitosterol/TC stratified by genotype groups for the 8 SNPs significantly associated with both markers are shown in Figures S7–S14.

### 3.2. Associations Unique to One Marker of Intestinal Cholesterol Absorption

Using the recessive model, the SNP (rs11051695) located in an intergenic region was identified to be associated with higher campesterol/TC but not sitosterol/TC levels.

On the other hand, 7 SNPs were associated with higher sitosterol/TC only, of which rs12977100 in the transmembrane and immunoglobulin domain containing 2 (*TMIGD2*) gene was identified under the additive model, while 6 SNPs were identified using the recessive model. Of these 6, 2 were located in protein-coding genes, which were rs7537876 in the formin 2 (*FMN2*) gene and rs1046345 in the FXYD domain containing ion transport regulator 5 (*FXYD5*) gene. The remaining 4 SNPs were in non-coding regions, including two intergenic SNPs (rs1925432 and rs66937025), and 2 SNPs within lnRNA (rs6471632 and rs2368283). All 8 SNPs reached suggestive significance. The corresponding β coefficients are presented in Table 3.

### 3.3. Linkage Disequilibrium (LD) and Selection of Tag SNPs for Associations Overlapping with Both Markers

**Figure 4:**
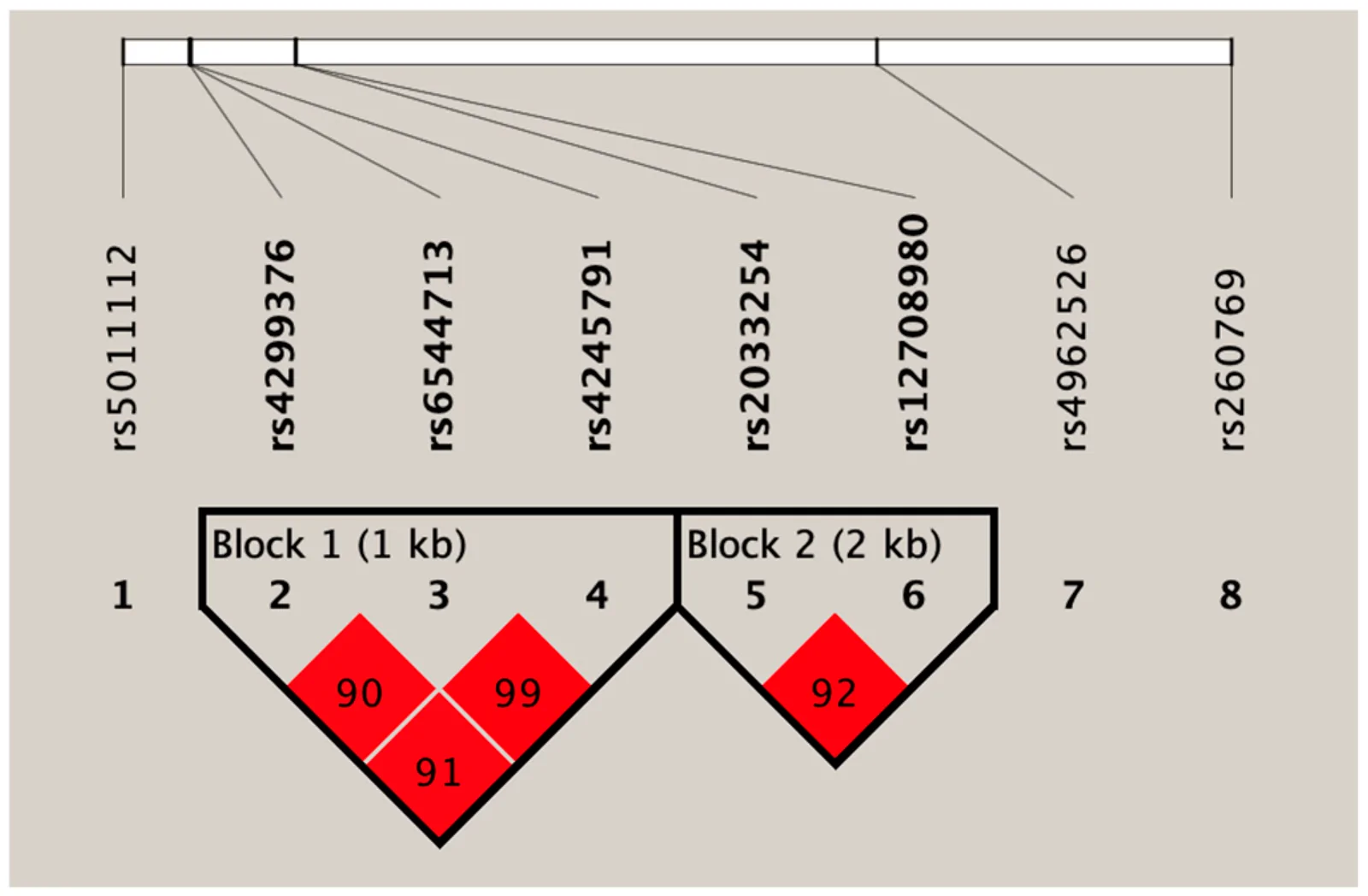
Haplotype blocks for the 8 SNPs that associated with campesterol/TC and sitosterol/TC. Linkage disequilibrium (LD) between the SNPs is denoted by the red squares, and the numbers refer to the r^2^ values. SNPs in bold constitute the haplotype blocks.

Two haplotype blocks were generated by Haploview (Figure 4). The first block comprised 3 SNPs in the *ABCG8* gene (rs4299376, rs6544713, and rs4245791; r^2^ ≥ 0.9 between all SNPs), while the second block comprised 2 SNPs in the *CETP* gene (rs2033254 and rs12708980; r^2^ = 0.92). Furthermore, 2 tag SNPs were identified: tag SNP rs4299376, which captured rs6544713 and rs4245791, and tag SNP rs2033254, which captured rs12708980.

## 4. Discussion

Although numerous genetic association studies have successfully identified SNPs associated with serum LDL-C concentrations and CVD risk [19,20,21,22], the relationship between SNPs and intestinal cholesterol absorption is largely unexplored. A previous meta-analysis has reported that a high intestinal cholesterol absorption is associated with increased CVD risk, independent of serum cholesterol concentrations [23]. Beyond CVD, intestinal cholesterol absorption has also been linked to other disorders, such as being higher with kidney diseases and lower with metabolic syndrome [24]. These observations highlight the importance of understanding associations between genetic variants and intestinal cholesterol absorption, which was examined in the present study in a European population. To reduce the likelihood of finding spurious associations, only the SNPs associated with both campesterol/TC and sitosterol/TC were considered relevant.

Only three of the identified loci were located within protein-coding genes. Given its well-known role in intestinal cholesterol absorption, *ABCG8* was an obvious candidate gene [25]. The tag SNP rs4299376 was associated with elevated levels of both campesterol/TC and sitosterol/TC. In line with this finding, this SNP has been repeatedly linked to higher levels of both markers for intestinal absorption in European populations, including cohorts from the LUdwisghafen RIsk and Cardiovascular Health Study (LURIC) and the Young Finns Study (YFS) [23], other population-based studies [26] and large-scale GWAS [27,28]. This association may also be reflected in elevated circulating LDL-C concentrations, as reported in other GWASs for the same SNP [29,30]. However, the molecular mechanism underlying this association has not yet been examined. Despite the well-recognized joint role of *ABCG8* with the *ABCG5* gene in intestinal cholesterol absorption and their overlap in the human genome, no SNPs in *ABCG5* were identified in this study. However, this does not exclude the contribution of *ABCG5* to inter-individual variations in cholesterol absorption, as previously reported [31].

Five additional novel SNPs were identified that were linked to intestinal cholesterol absorption markers. Both *ADAM12* and *CETP* genes are expressed in the small intestine [32]. However, it is currently unclear how variations in the *ADAM12* gene might affect cholesterol absorption, as it has not been previously implicated in cholesterol metabolism. In contrast, *CETP* is known for its role in lipid transfer across lipoproteins [33]. Although a direct role of CETP in intestinal cholesterol absorption has not been demonstrated, several intervention studies suggest a link with plant sterol metabolism. Genetic variation in *CETP* has been associated with interindividual differences in the response to plant sterol consumption, including greater TG-lowering and genotype-associated changes in LDL-C and circulating CETP concentrations following plant sterol intake [34,35]. In addition, plant stanol consumption has been reported to reduce circulating CETP mass [36]. Together, these findings suggest that CETP may influence physiological responses to plant sterol or stanol interventions. However, the underlying mechanisms remain unclear, and further studies are needed to determine whether CETP also plays a direct role in intestinal cholesterol absorption.

Several identified SNPs mapped to non-coding regions. Variants in such regions may still be functional, as the majority of SNPs identified by the GWAS that are associated with complex traits are located in non-coding regions [37]. This may be explained by regulatory effects on nearby coding genes or by LD with nearby variants in coding regions [37]. Nevertheless, the physiological roles of non-coding regions, such as intergenic sequences or non-coding RNAs, need further study.

To explore the potential functional impact of the identified SNPs, in silico pathogenicity prediction tools available in Ensembl (release 115) were used [38], including Polymorphism Phenotyping (PolyPhen-2), Rare Exome Variant Ensemble Learner (REVEL) scores, Sorting Intolerant From Tolerant (SIFT), and Combined Annotation Dependent Depletion (CADD) scores [39,40,41,42]. However, most of these scores are primarily applicable to missense variants or to variants in protein-coding regions and were therefore not informative for the variants identified in this study, as none were predicted to result in amino acid substitutions. Potential splice effects were assessed using SpliceAI. [43], which predicted no impact on splicing for any of the SNPs. Additionally, we examined whether any of the identified SNPs could alter gene expression in the small intestine. However, no significant expression quantitative trait loci (eQTLs) were identified through public databases [32]. Further, we assessed through Ensembl whether any of the identified SNPs were in LD with other SNPs in genes known to be involved in intestinal cholesterol absorption. Based on the currently available European reference database, no such relationships were identified [38]. Moreover, a protein–protein interaction network analysis could have provided meaningful insights into how the genes identified in this study interact within biological pathways related to intestinal cholesterol absorption. However, this analysis was not pursued, as the limited number of genes identified was insufficient to generate a meaningful network or yield additional biological insights. Furthermore, no functional studies have investigated the SNPs identified in this study in relation to intestinal cholesterol absorption. Taken together, the mechanisms underlying the observed associations remain unclear.

To our knowledge, no previous GWAS have investigated genetic associations with TC-standardized campesterol and sitosterol levels as markers of intestinal cholesterol absorption. Therefore, direct comparison with previous studies is limited. Notably, variants within *CETP* and *ADAM12* have not previously been implicated in intestinal cholesterol absorption, suggesting that these loci may represent novel candidates for further study. The identification of SNPs in loci with previously unrecognized potential roles in intestinal cholesterol absorption provides new opportunities for further research.

However, several limitations should be considered. First, the sample size of this study was relatively modest, which may have limited the ability to detect genetic variants with small effect sizes due to low statistical power. Furthermore, recessive analyses were exploratory and restricted to variants with at least 30 minor allele homozygotes. Consequently, recessive effects of less frequent variants may have been missed. Second, pooling participants from five independent studies may have introduced residual heterogeneity, although this was addressed by including ‘Study’ as a covariate in all analyses. Third, the identified associations have not yet been replicated. Therefore, these findings should be interpreted with caution and require validation in larger, independent populations before firm conclusions can be drawn. Finally, the observed associations do not establish causality. Further functional and mechanistic studies are needed to elucidate underlying biological mechanisms and their potential role in intestinal cholesterol absorption.

## 5. Conclusions

This GWAS confirmed previously reported associations within *ABCG8* and identified candidate loci in *CETP* and *ADAM12* that may be involved in intestinal cholesterol absorption. These findings contribute to our understanding of genetic factors underlying intestinal cholesterol absorption and highlight candidate loci for future replication. Functional studies are required to elucidate the potential roles of these loci in intestinal cholesterol absorption.

## Figures and Tables

**Table 1:** Genomic annotation, genotype frequencies, allele frequencies, and genotype call rates for the 16 SNPs associated with campesterol/TC and/or sitosterol/TC in the study population (N = 398).

Chr	POS	rsID	HGVS Nomenclature	Gene (HGNC Symbol)	Consequence	Ref/Alt	Genotype Frequency N (%)			Allele Frequency (%)		Call Rate (%)
							WT	Het	Hom	RAF	MAF	
1	240303322	rs7537876	NC_000001.10:g.240303322C>T	*FMN2* (HGNC:14074)	Intron	C/T	109 (27)	208 (52)	80 (20)	54	46	99.7
2	44072576	rs4299376	NC_000002.11:g.44072576G>T	*ABCG8* (HGNC:13887)	Intron	G/T	168 (43)	192 (49)	32 (8)	67	33	98.5
2	44073881	rs6544713	NC_000002.11:g.44073881T>C	*ABCG8* (HGNC:13887)	Intron	T/C	180 (45)	186 (47)	32 (8)	69	31	100
2	44074431	rs4245791	NC_000002.11:g.44074431C>T	*ABCG8* (HGNC:13887)	Intron	C/T	180 (45)	185 (46)	33 (8)	68	32	100
3	171251343	rs260769	NC_000003.11:g.171251343T>C	*-*	Intergenic	T/C	183 (46)	171 (43)	44 (11)	67	33	100
4	35751033	rs5011112	NC_000004.11:g.35751033T>C	*-*	Intergenic	T/C	205 (52)	159 (40)	34 (9)	71	29	100
6	23294008	rs1925432	NC_000006.11:g.23294008T>C	*-*	Intergenic	T/C	164 (41)	185 (47)	48 (12)	65	35	99.7
8	58486824	rs6471632	NC_000008.10:g.58486824G>A	*lncRNA*	Intron	G/A	159 (40)	187 (47)	52 (13)	63	37	100
10	28311172	rs2368283	NC_000010.10:g.28311172A>G	*lncRNA*	Intron	A/G	228 (57)	138 (35)	32 (8)	75	25	100
10	127929499	rs4962526	NC_000010.10:g.127929499A>G	*ADAM12* (HGNC:190)	Intron	A/G	197 (49)	170 (43)	31 (8)	71	29	100
12	32100474	rs11051695	NC_000012.11:g.32100474G>A	*-*	Intergenic	G/A	143 (36)	199 (50)	55 (14)	61	39	99.7
16	57009985	rs2033254	NC_000016.9:g.57009985T>C	*CETP* (HGNC:1869)	Intron	T/C	168 (42)	189 (47)	41 (10)	66	34	100
16	57012379	rs12708980	NC_000016.9:g.57012379T>G	*CETP* (HGNC:1869)	Intron	T/G	159 (40)	193 (48)	46 (12)	64	36	100
18	70348463	rs66937025	NC_000018.9:g.70348463C>T	*-*	Intergenic	C/T	109 (28)	213 (54)	74 (19)	54	46	99.5
19	4294542	rs12977100	NC_000019.9:g.4294542C>T	*TMIGD2* (HGNC:28324)	Intron	C/T	242 (61)	132 (33)	24 (6)	77	23	100
19	35660752	rs1046345	NC_000019.9:g.35660752C>T	*FXYD5* (HGNC:4029)	3 prime UTR	C/T	143 (36)	190 (48)	65 (16)	60	40	100

**Abbreviations:** Chr, chromosome; POS, position; rsID, reference SNP cluster identifier; HGVS, The Human Genome Variation Society; HGNC, The HUGO Gene Nomenclature Committee; Ref/Alt, reference/alternative alleles; Genotype frequency (WT, wild-type; Het, heterozygotes; Hom, homozygotes for the minor (alternative) allele); RAF, reference allele frequency; MAF, minor (alternative) allele frequency. Genes full names: *FMN2* (HGNC:14074), formin 2; *ABCG8* (HGNC:13887), ATP binding cassette subfamily G member 8; *lncRNA*, Long non-coding RNA; *ADAM12* (HGNC:190), ADAM metallopeptidase domain 12; *CETP* (HGNC:1869), cholesteryl ester transfer protein; *TMIGD2* (HGNC:28324), transmembrane and immunoglobulin domain containing 2; *FXYD5* (HGNC:4029), FXYD domain containing ion transport regulator 5. SNPs’ positions and HGVS nomenclature are in GRCh37 coordinates. Consequences of SNPs were obtained from Ensembl.

**Table 2:** SNPs reaching genome-wide or suggestive significance for both campesterol/total cholesterol (TC) and sitosterol/TC.

Chr	rsID	HGVS Nomenclature	Gene (HGNC Symbol)	Ref/Alt	N	Trait	Model	β	95% CI	*p*
2	rs4299376	NC_000002.11:g.44072576G>T	*ABCG8* (HGNC:13887)	G/T	392	Camp/TC	ADD	39.2	25.7 to 52.7	**2.76 × 10^−8^**
						Sit/TC	ADD	25.4	16.6 to 34.1	**2.51 × 10^−8^**
2	rs6544713	NC_000002.11:g.44073881T>C	*ABCG8* (HGNC:13887)	T/C	398	Camp/TC	ADD	37.3	23.9 to 50.7	8.10 × 10^−8^
						Sit/TC	ADD	24.3	15.6 to 32.9	6.67 × 10^−8^
2	rs4245791	NC_000002.11:g.44074431C>T	*ABCG8* (HGNC:13887)	C/T	398	Camp/TC	ADD	37.4	24.1 to 50.7	6.25 × 10^−8^
						Sit/TC	ADD	24.3	15.7 to 32.9	5.32 × 10^−8^
3	rs260769 *	NC_000003.11:g.171251343T>C	*-*	T/C	398	Camp/TC	REC	68.7	41.8 to 95.6	8.25 × 10^−7^
						Sit/TC	REC	50.0	32.8 to 67.3	**2.36 × 10^−8^**
4	rs5011112 *	NC_000004.11:g.35751033T>C	*-*	T/C	398	Camp/TC	REC	74.7	44.3 to 105.2	2.18 × 10^−6^
						Sit/TC	REC	52.6	33.0 to 72.1	2.31 × 10^−7^
10	rs4962526 *	NC_000010.10:g.127929499A>G	*ADAM12* (HGNC:190)	A/G	398	Camp/TC	REC	78.4	46.9 to 109.9	1.57 × 10^−6^
						Sit/TC	REC	54.1	33.9 to 74.4	2.62 × 10^−7^
16	rs2033254 *	NC_000016.9:g.57009985T>C	*CETP* (HGNC:1869)	T/C	398	Camp/TC	ADD	−32.5	−45.4 to −19.5	1.34 × 10^−6^
						Sit/TC	ADD	−21.0	−29.3 to −12.6	1.37 × 10^−6^
16	rs12708980 *	NC_000016.9:g.57012379T>G	*CETP* (HGNC:1869)	T/G	398	Camp/TC	ADD	−29.6	−42.5 to −16.8	8.61 × 10^−6^
						Sit/TC	ADD	−19.5	−27.8 to −11.2	5.75 × 10^−6^

**Abbreviations:** Chr, chromosome; rsID, reference SNP cluster identifier; HGVS, The Human Genome Variation Society; HGNC, The HUGO Gene Nomenclature Committee; Ref/Alt, reference/alternative alleles; N, total number of observations (used in the analysis); Camp/TC, TC-standardized campesterol; Sit/TC, TC-standardized sitosterol; Model (ADD, additive; REC, recessive). Genes full names: *ABCG8* (HGNC:13887), ATP binding cassette subfamily G member 8; *ADAM12* (HGNC:190), ADAM metallopeptidase domain 12; *CETP* (HGNC:1869), cholesteryl ester transfer protein. HGVS nomenclature is in GRCh37 coordinates. The tested (effect) allele is the alternative allele as shown in the table. The β coefficients represent the estimated change in 10^2^ × μmol/mmol of the marker with each copy of the alternative allele relative to the reference allele for the additive model and the estimated change in 10^2^ × μmol/mmol of the marker for individuals homozygous for the alternative allele compared with individuals with at least one reference allele for the recessive model. SNPs with their *p* values in bold represent genome-wide significance (*p* ≤ 5 × 10^−8^), while non-bold values represent associations with suggestive significance (*p* ≤ 1 × 10^−5^). * 5 SNPs not reported before.

**Table 3:** SNPs associated with either serum campesterol/TC or sitosterol/TC levels under additive and recessive models.

Chr	rsID	HGVS Nomenclature	Gene (HGNC Symbol)	Ref/Alt	N	Model	β	95% CI	*p*
SNPs associated with campesterol/TC									
12	rs11051695	NC_000012.11:g.32100474G>A	*-*	G/A	397	REC	56.8	32.0 to 81.5	9.35 × 10^−6^
SNPs associated with sitosterol/TC									
1	rs7537876	NC_000001.10:g.240303322C>T	*FMN2* (HGNC:14074)	T/C	397	REC	31.5	17.7 to 45.3	9.57 × 10^−6^
6	rs1925432	NC_000006.11:g.23294008T>C	*-*	T/C	397	REC	40.4	23.3 to 57.5	4.88 × 10^−6^
8	rs6471632	NC_000008.10:g.58486824G>A	*lncRNA*	G/A	398	REC	37.7	21.4 to 54.1	8.18 × 10^−6^
10	rs2368283	NC_000010.10:g.28311172A>G	*lncRNA*	G/A	398	REC	48.3	27.9 to 68.6	4.60 × 10^−6^
18	rs66937025	NC_000018.9:g.70348463C>T	*-*	T/C	396	REC	32.3	18.2 to 46.4	9.06 × 10^−6^
19	rs12977100	NC_000019.9:g.4294542C>T	*TMIGD2* (HGNC:28324)	T/C	398	ADD	21.4	12.4 to 30.4	4.28 × 10^−6^
19	rs1046345	NC_000019.9:g.35660752C>T	*FXYD5* (HGNC:4029)	T/C	398	REC	35.7	20.7 to 50.6	4.16 × 10^−6^

**Abbreviations:** Chr, chromosome; rsID, reference SNP cluster identifier; HGVS, The Human Genome Variation Society; HGNC, The HUGO Gene Nomenclature Committee; Ref/Alt, reference/alternative alleles; N, total number of observations (used in the analysis); Model (ADD, additive; REC, recessive). Genes full names: *FMN2* (HGNC:14074), formin 2; *lncRNA*, Long non-coding RNA; *TMIGD2* (HGNC:28324), Transmembrane and immunoglobulin domain containing 2; *FXYD5* (HGNC:4029), FXYD domain containing ion transport regulator 5. HGVS nomenclature is in GRCh37 coordinates. The tested (effect) allele is the alternative allele as shown in the table. The β coefficients represent the estimated change in 10^2^ × μmol/mmol of the marker with each copy of the alternative allele relative to the reference allele for the additive model and the estimated change in 10^2^ × μmol/mmol of the marker for individuals homozygous for the alternative allele compared with individuals with at least one reference allele for the recessive model. All *p* values indicate associations reaching suggestive significance (*p* ≤ 1 × 10^−5^).

## Data Availability

The dataset presented in this article is not readily available because data sharing is subject to ethical and data protection regulations. Data may be made available from the principal investigator upon reasonable request and subject to approval by the relevant ethics committee and data protection requirements.

## Supplementary Materials

The following supporting information can be downloaded at https://www.mdpi.com/article/10.3390/nu18162679/s1, Table S1: Baseline characteristics of the study participants; Table S2: TC-standardized campesterol and sitosterol levels by genotype groups for the 16 identified SNPs; Figure S1: Histograms of HWE *p*-values of all SNPs; Figure S2: Histogram of heterozygosity rate of the cohort; Figure S3: Scree plot for principal components; Figure S4: Visualization of the first two principal components (PCs); Figure S5: Histogram of minor allele frequencies (MAF) before filtering out SNPs at a MAF threshold of 20%; Figure S6: Quantile-quantile (Q-Q) plot for associations with serum campesterol/TC and sitosterol/TC using additive and recessive models; Figures S7–S14: Boxplots of campesterol/TC and sitosterol/TC stratified by genotype groups for the 8 SNPs significantly associated with both markers.

## Abbreviations

The following abbreviations are used in this manuscript:

ABC	ATP-binding cassette
ABCG5	ATP-binding cassette subfamily G member 5
ABCG8	ATP-binding cassette subfamily G member 8
ADAM12	ADAM metallopeptidase domain 12
ANOVA	Analysis of variance
azM	Academic Hospital Maastricht (Academisch ziekenhuis Maastricht)
BMI	Body mass index
CETP	Cholesteryl ester transfer protein
CVD	Cardiovascular disease
DNA	Deoxyribonucleic acid
eQTL	Expression quantitative trait locus
FMN2	Formin 2
FXYD5	FXYD domain containing ion transport regulator 5
GC-FID	Gas chromatography with flame-ionization detection
GC-MS	Gas chromatography–mass spectrometry
GWAS	Genome-wide association study
HWE	Hardy–Weinberg equilibrium
kb	Kilobase
LD	Linkage disequilibrium
LDL-C	Low-density lipoprotein cholesterol
lncRNA	Long non-coding RNA
LOD	Logarithm of odds
LURIC	LUdwigshafen RIsk and Cardiovascular Health Study
MAF	Minor allele frequency
METC	Medical Ethical Committee
NPC1L1	Niemann–Pick C1-Like 1
PC	Principal component
PCA	Principal component analysis
PI_HAT	Proportion of identity by descent
PLINK	Whole-genome association analysis toolset
PMRA	Precision Medicine Research Array
PS	Plant sterols and stanols
Q-Q	Quantile–quantile
QC	Quality control
RNA	Ribonucleic acid
SD	Standard deviation
SNP	Single nucleotide polymorphism
SpliceAI	Splicing prediction using artificial intelligence
TC	Total cholesterol
TICE	Transintestinal cholesterol excretion
TMIGD2	Transmembrane and immunoglobulin domain containing 2
UM	Maastricht University
YFS	Young Finns Study
β	Beta coefficient
λ^GC^	Genomic inflation factor
