# Supplementary material for: Genome-Wide Association of Genetic Variants with Intestinal Cholesterol Absorption Markers in a European Population
Source: Nutrients. 2026 Aug 16;18(16):2679. doi: 10.3390/nu18162679 (PMC13516138; doi:10.3390/nu18162679)
Supplement: Supplementary file 1 [file nutrients-18-02679-s001.zip › nutrients-4420146-supplementary.pdf]

Supplementary material for

Genome-wide association of genetic variants with intestinal cholesterol absorption markers in a European population

Fatma B. A. Mokhtar [1,†], Dena A. Nuwaylati [1,2,†], Jogchum Plat [1*], Susan L.M. Coort [3], Herman E. Popeijus [1], Marcus E. Kleber [4,5], Dieter Lütjohann [6], Ronald P. Mensink [1]

[†] These authors contributed equally to this work and share first authorship.

Authors' affiliations:

[1] Department of Nutrition and Movement Sciences, NUTRIM Institute of Nutrition and Translational Research in Metabolism, Maastricht University, 6200 MD, Maastricht, The Netherlands; fatma.bashir31@gmail.com (F.B.M); dena.nuwaylati@maastrichtuniversity.nl (D.A.N); j.plat@maastrichtuniversity.nl (J.P); h.popeijus@maastrichtuniversity.nl (H.E.P); r.mensink@maastrichtuniversity.nl (R.P.M)

[2] Division of Clinical Biochemistry, Department of Basic Medical Sciences, College of Medicine, University of Jeddah, Jeddah 23890, Saudi Arabia.

[3] Department of Translational Genomics, NUTRIM Institute of Nutrition and Translational Research in Metabolism, Maastricht University, 6200 MD, Maastricht, The Netherlands; susan.coort@maastrichtuniversity.nl (S.C)

[4] Department of Medicine I (Cardiology, Hemostaseology, Medical Intensive Care), Medical Faculty Mannheim, Heidelberg University, 68167 Mannheim, Germany; Marcus.Kleber@medma.uni-heidelberg.de (M.E.K)

[5] SYNLAB MVZ Humangenetik Mannheim, Mannheim, Baden-Württemberg, 68163 Germany

[6] Institute of Clinical Chemistry and Clinical Pharmacology, University Hospital Bonn, Venusberg-Campus 1, 53127 Bonn, Germany; Dieter.Luetjohann@ukbonn.de (D.L)

Content:

- **Table S1.** Baseline characteristics of the study participants.
- **Table S2.** TC-standardized campesterol and sitosterol levels by genotype groups for the 16 identified SNPs.
- **Figure S1.** Histograms of HWE p-values of all SNPs.
- **Figure S2.** Histogram of heterozygosity rate of the cohort.
- **Figure S3.** Scree plot for principal components.
- **Figure S4.** Visualization of the first two principal components (PCs).
- **Figure S5.** Histogram of minor allele frequencies (MAF) before filtering out SNPs at a MAF threshold of 20%.
- **Figure S6.** Quantile-quantile (Q-Q) plot for associations with serum campesterol/TC and sitosterol/TC using additive and recessive models.

- **Figures S7-S14.** Boxplots of campesterol/TC and sitosterol/TC stratified by genotype groups for the 8 SNPs significantly associated with both markers

Table S1. Baseline characteristics of the study participants

	Study 1	Study 2	Study 3	Study 4	Study 5	Total	*P*
N	97	18	31	234	18	398	
Age (years)	32.8 ± 15.0	26.7 ± 11.8	50.5 ± 12.9	53.0 ± 10.1	33.0 ± 12.2	45.8 ± 15.4	< 0.001
Sex (M/F) n(%)	35 (36.1) / 62 (63.9)	4 (22.2) / 14 (77.8)	13 (41.9) / 18 (58.1)	118 (50.4) / 116 (49.6)	7 (38.9) / 11 (61.1)	177 (44.5) / 221 (55.5)	0.040
BMI (kg/m2)	23.1 ± 2.9	22.7 ± 2.7	25.6 ± 3.1	26.4 ± 3.5	23.9 ± 2.8	25.2 ± 3.6	< 0.001
Smokers (%)*	18 (18.6)	NA	3 (9.7)	30 (12.8)	0 (0)	51 (13.0)	< 0.001
Lipids (mmol/L)							
TC	4.95 ± 0.79	4.49 ± 0.71	6.00 ± 0.85	5.72 ± 1.01	5.38 ± 0.83	5.48 ± 1.01	< 0.001
LDL-C	2.93 ± 0.76	2.62 ± 0.67	4.02 ± 0.83	3.80 ± 0.86	3.20 ± 0.76	3.52 ± 0.93	< 0.001
HDL-C	1.60 ± 0.37	1.42 ± 0.23	1.40 ± 0.44	1.40 ± 0.43	1.59 ± 0.33	1.46 ± 0.41	< 0.001
TAG	0.81 (0.73-0.89)	0.92 (0.76-1.11)	1.11 (0.92-1.33)	1.01 (0.95-1.07)	1.25 (1.07-1.46)	0.97 (0.92 - 1.02)	< 0.001
Serum NCS (10^2×µmol/mmol)							
Campesterol/TC	317 ± 101	242 ± 99	239 ± 67	153 ± 86	249 ± 92	208 ± 113	< 0.001
Sitosterol/TC	120 ± 46	159 ± 57	156 ± 37	134 ± 63	155 ± 60	134 ± 58	0.004

Abbreviations: BMI, body mass index; TC, total cholesterol; LDL-C, low-density lipoprotein cholesterol; HDL-C, high-density lipoprotein cholesterol; TAG, triacylglycerol; NCS, non-cholesterol sterols; NA, not available. Data are presented as mean ± SD, or n(%) (for sex and smokers). TAG values are presented as mean (95% CI) of the back-transformed values after the log-transformation. P values for differences between the studies were obtained using ANOVA or chi square. *Data were available for n=380.

Table S2. TC-standardized campesterol and sitosterol levels by genotype groups for the 16 identified SNPs

rsID	Ref/Alt	Alleles	N	Campesterol/TC (10^2×µmol/mmol)			Sitosterol/TC (10^2×µmol/mmol)		
				**Mean ± SD**	**Min**	**Max**	**Mean ± SD**	**Min**	**Max**
rs7537876	C/T	**CC**	109	198 ± 96	35	463	125 ± 40	48	291
		**CT**	208	202 ± 107	28	701	129 ± 47	43	326
		**TT**	80	236 ± 144	59	675	161 ± 91	44	520
rs4299376	G/T	**GG**	168	185 ± 100	28	512	119 ± 44	43	326
		**GT**	192	217 ± 112	35	675	142 ± 61	44	511
		**TT**	32	289 ± 149	47	701	174 ± 79	79	520
rs6544713	T/C	**TT**	180	185 ± 99	28	512	120 ± 44	43	326
		**TC**	186	217 ± 112	35	675	142 ± 62	44	511
		**CC**	32	286 ± 152	47	701	172 ± 80	79	520
rs4245791	C/T	**CC**	180	185 ± 99	28	512	120 ± 44	43	326
		**CT**	185	217 ± 112	35	675	142 ± 62	44	511
		**TT**	33	283 ± 150	47	701	172 ± 79	79	520
rs260769	T/C	**TT**	183	207 ± 110	39	701	130 ± 51	47	511
		**TC**	171	192 ± 98	28	538	127 ± 44	43	291
		**CC**	44	276 ± 153	35	675	179 ± 101	51	520
rs5011112	T/C	**TT**	205	202 ± 104	28	675	133 ± 49	48	352
		**TC**	159	199 ± 107	32	538	125 ± 45	43	326
		**CC**	34	285 ± 163	92	701	180 ± 116	58	520
rs1925432	T/C	**TT**	164	197 ± 105	38	554	128 ± 47	44	345
		**TC**	185	205 ± 106	28	701	132 ± 50	43	352
		**CC**	48	258 ± 153	39	675	162 ± 102	71	520
rs6471632	G/A	**GG**	159	207 ± 116	55	701	128 ± 48	44	303
		**GA**	187	201 ± 106	28	554	130 ± 46	43	345
		**AA**	52	239 ± 130	57	624	169 ± 100	71	520
rs2368283	A/G	**AA**	228	209 ± 111	28	675	130 ± 47	43	303
		**AG**	138	195 ± 103	35	624	131 ± 55	58	511
		**GG**	32	259 ± 159	61	701	178 ± 106	59	520
rs4962526	A/G	**AA**	197	200 ± 107	36	603	133 ± 54	47	475
		**AG**	170	205 ± 101	28	538	127 ± 45	43	326

		**GG**	31	278 ± 179	64	701	182 ± 107	70	520
rs11051695	G/A	**GG**	143	204 ± 108	28	675	127 ± 43	43	303
		**GA**	199	199 ± 103	38	554	131 ± 49	44	352
		**AA**	55	254 ± 149	36	701	163 ± 101	47	520
rs2033254	T/C	**TT**	168	230 ± 133	28	701	149 ± 72	50	520
		**TC**	189	197 ± 95	40	428	127 ± 43	44	326
		**CC**	41	169 ± 87	32	368	108 ± 35	43	187
rs12708980	T/G	**TT**	159	227 ± 133	28	701	147 ± 73	50	520
		**TG**	193	201 ± 98	40	512	129 ± 45	44	326
		**GG**	46	172 ± 87	32	368	109 ± 35	43	187
rs66937025	C/T	**CC**	109	217 ± 118	38	675	132 ± 56	48	475
		**CT**	213	192 ± 96	28	528	127 ± 45	43	352
		**TT**	74	240 ± 144	40	701	158 ± 84	58	520
rs12977100	C/T	**CC**	242	195 ± 107	28	701	125 ± 47	43	303
		**CT**	132	227 ± 119	35	603	146 ± 66	47	520
		**TT**	24	233 ± 130	64	624	166 ± 91	84	511
rs1046345	C/T	**CC**	143	182 ± 96	32	538	129 ± 45	43	352
		**CT**	190	210 ± 109	28	701	130 ± 47	44	326
		**TT**	65	260 ± 142	56	675	160 ± 96	53	520

Abbreviations: rsID, reference SNP cluster Identifier; Ref/Alt, reference/alternative alleles; SD, standard deviation; Min, minimum; Max, maximum.

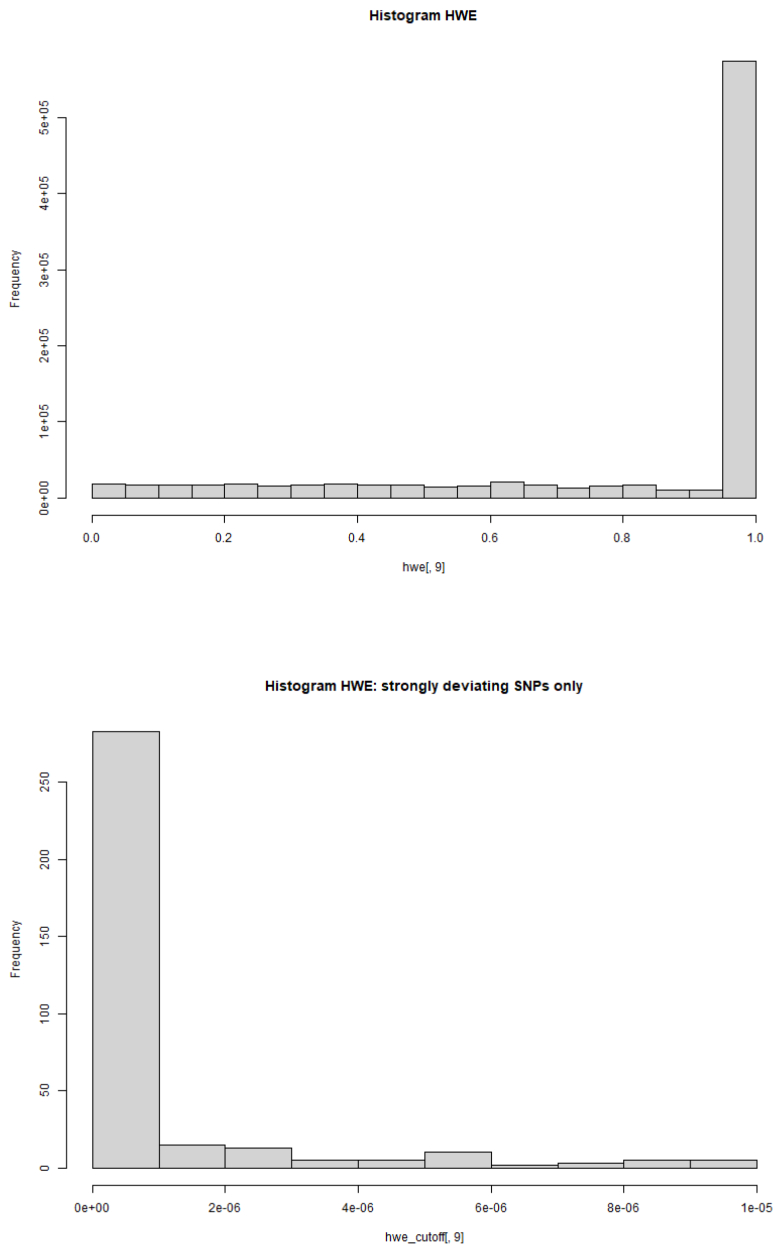

Figure S1. Histograms of HWE *p*-values of all SNPs (top) and SNPs with strong deviation (bottom) (n=421).

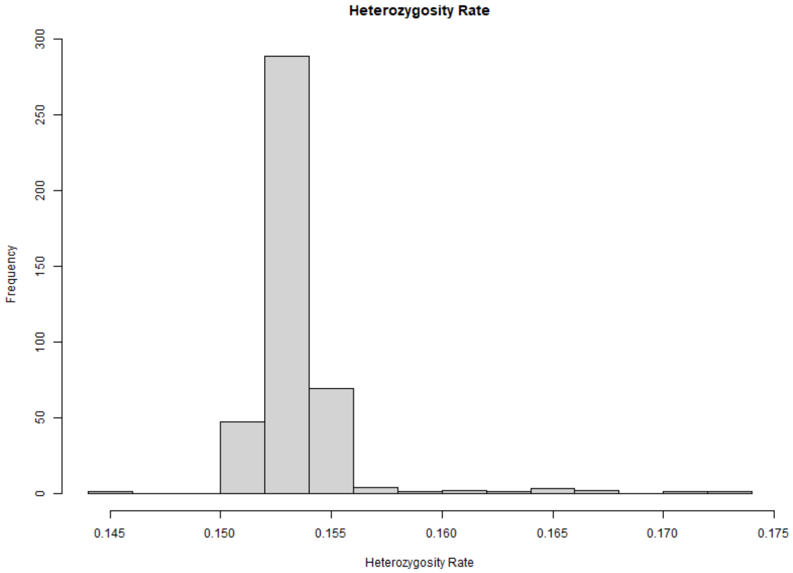

Figure S2. Histogram of heterozygosity rate of the cohort (n=421).

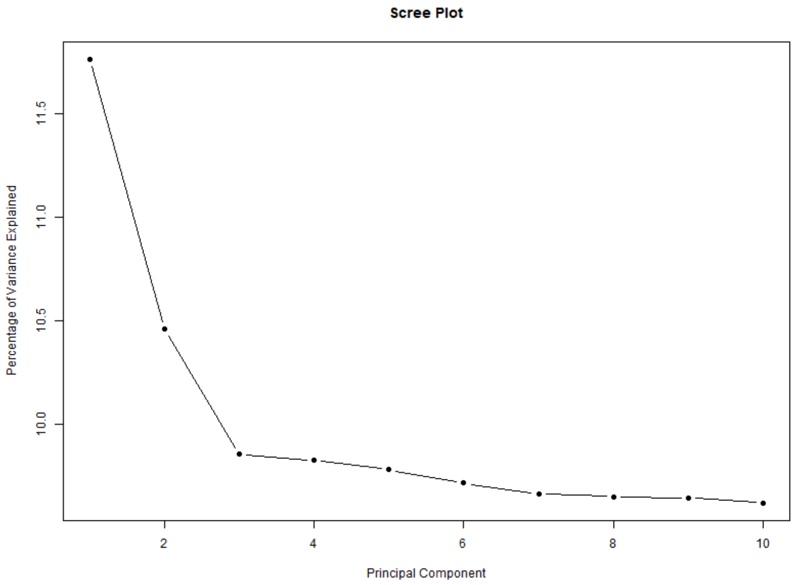

Figure S3. Scree plot of principal component analysis (n=410). The first 10 principal components (PCs) are on the x-axis, and their corresponding eigenvalues are on the y-axis. PC1 and PC2 were visually determined as the most relevant points of inflection.

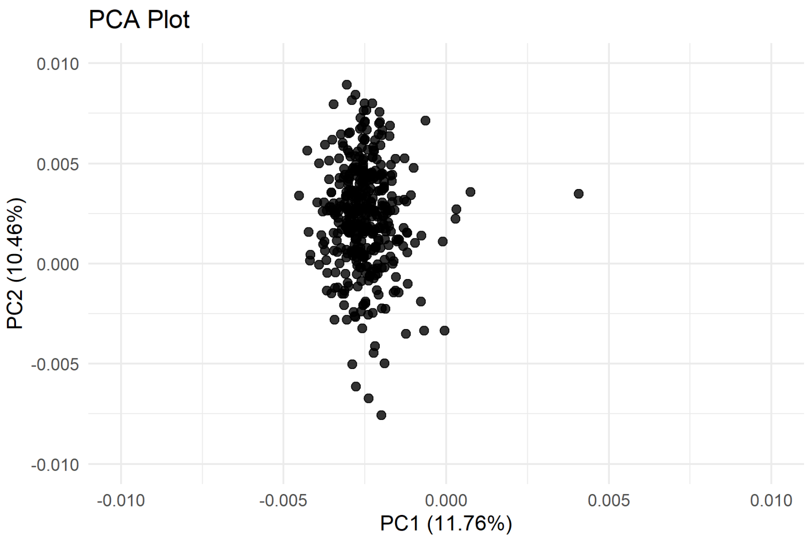

Figure S4. Visualization of the first two principal components (PCs) (n=410). PC1 explained 11.8% and PC2 explained 10.5% of the variance in the study population.

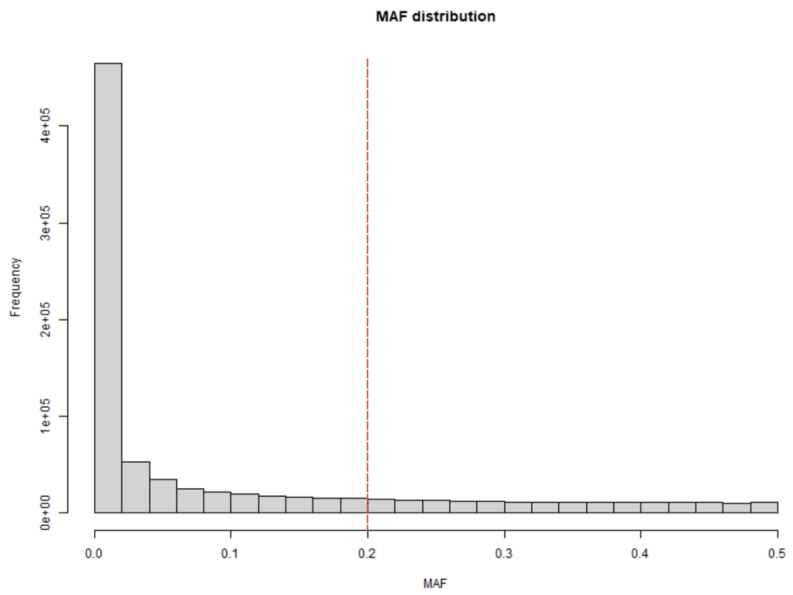

Figure S5. Histogram of minor allele frequencies (MAF) before filtering out SNPs at a MAF threshold of 20%. Frequency on the Y axis denotes the number of SNPs within each MAF group (n=410).

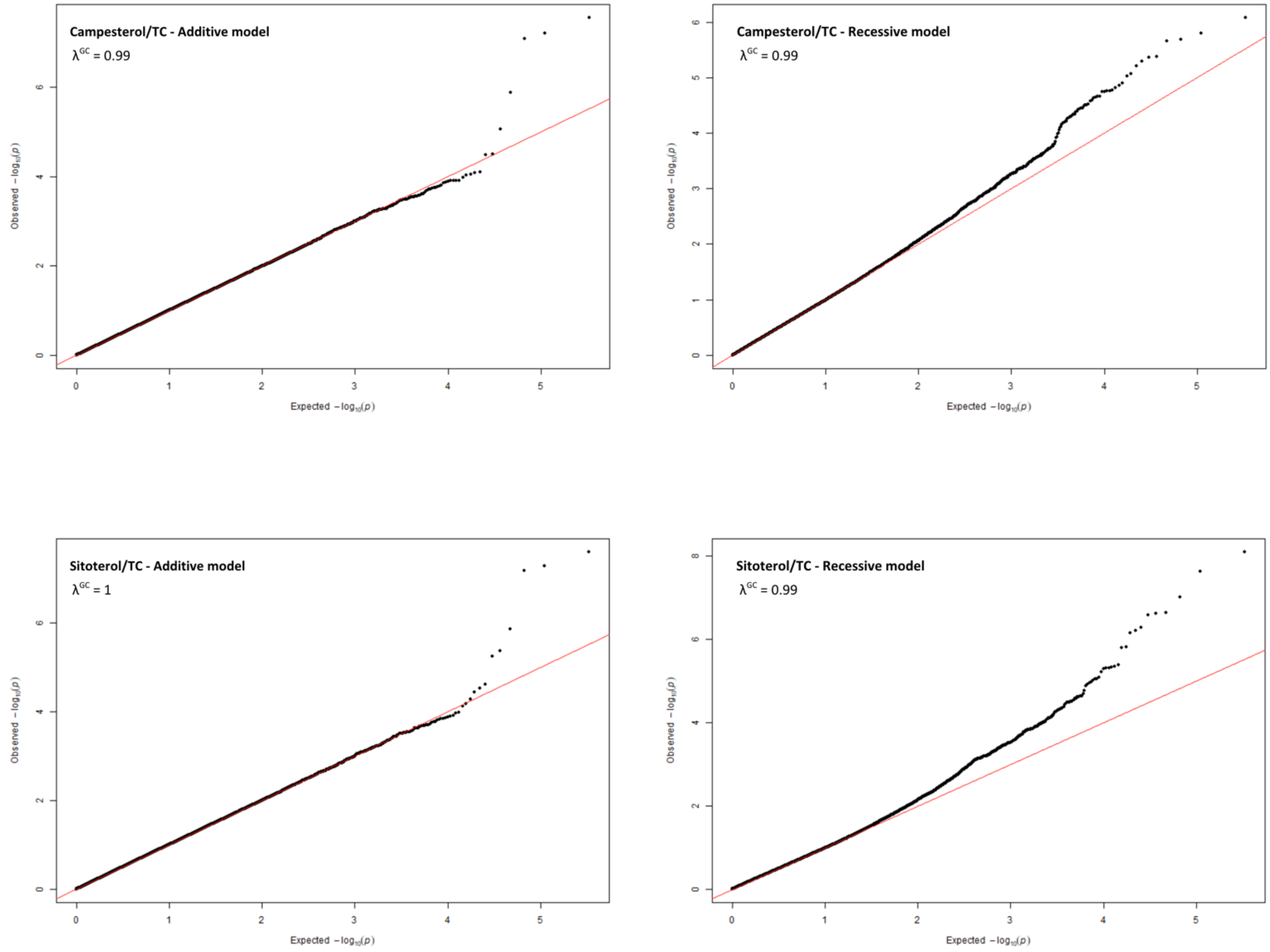

Figure S6. Quantile-quantile (Q-Q) plot for associations with serum campesterol/TC (top) and sitosterol/TC (bottom) using additive and recessive models (n=398). The redline represent the expected distribution, while the black dots are GWAS estimates accounted for population structure. The observed versus expected (χ^2) test statistics show no evidence of genomic inflation as reflected by genomic inflation factor (λ^{GC}) values calculated from the χ^2 statistics that were close to 1.

Boxplots of campesterol/TC and sitosterol/TC stratified by genotype groups for the 8 SNPs significantly associated with both markers

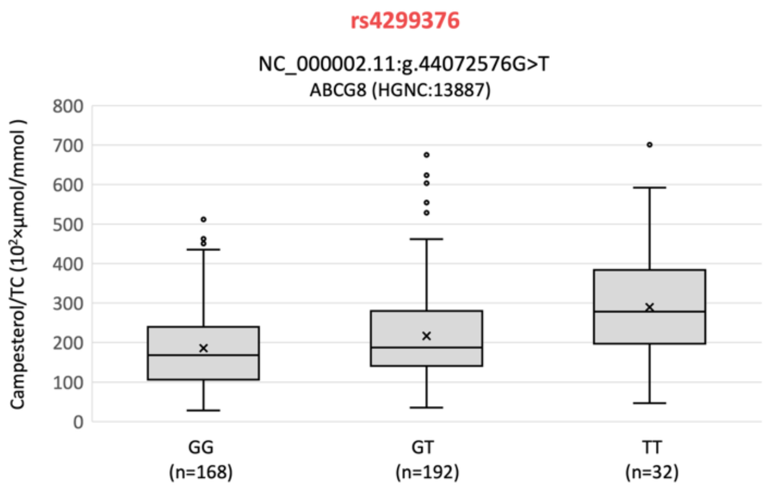

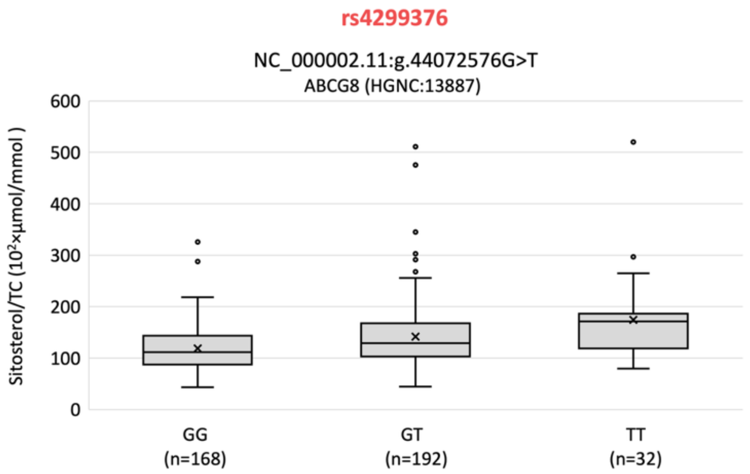

Figure S7. Boxplots for rs4299376

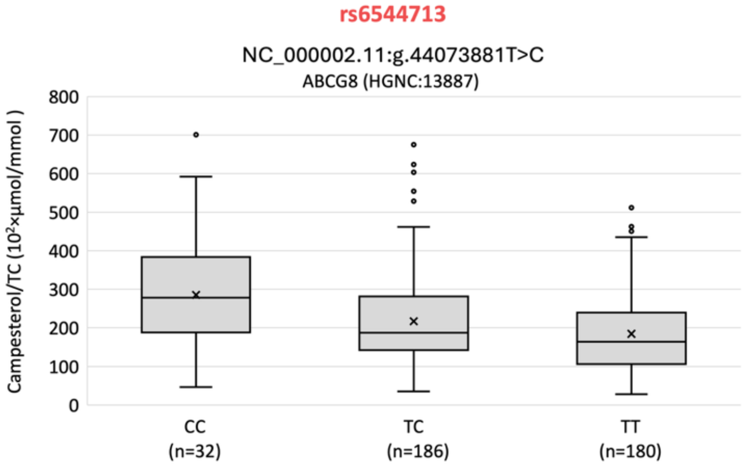

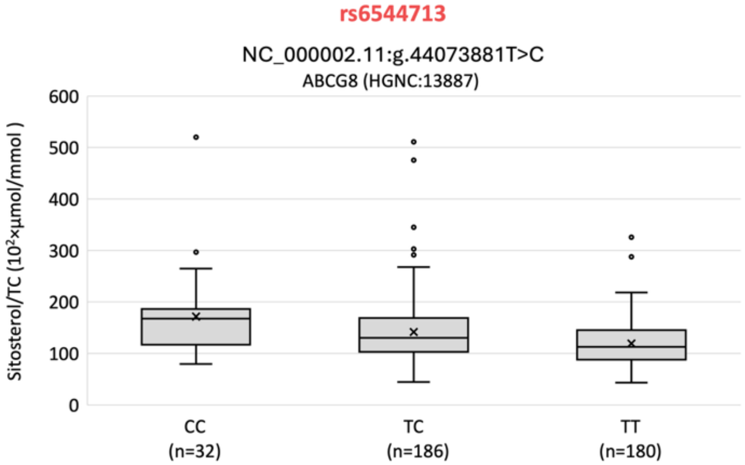

Figure S8. Boxplots for rs6544713

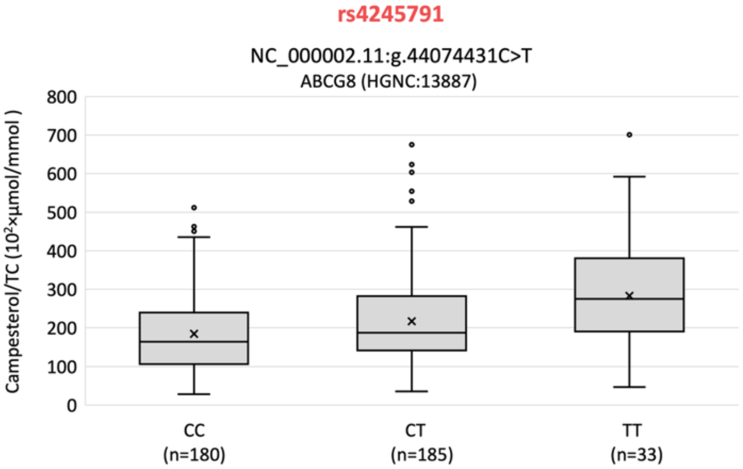

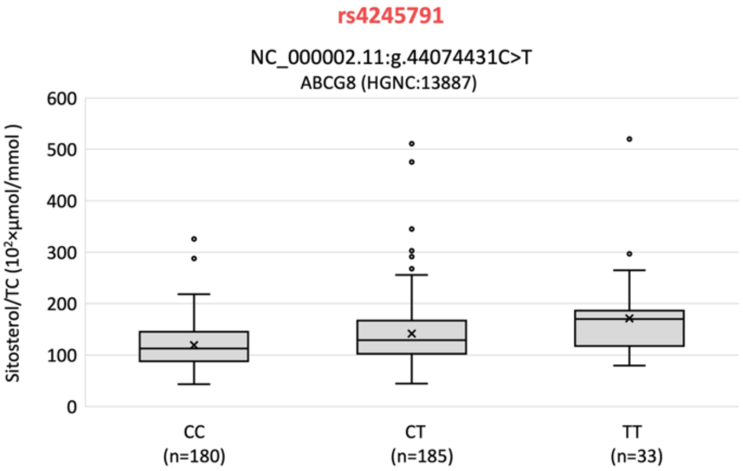

Figure S9. Boxplots for rs4245791

rs260769

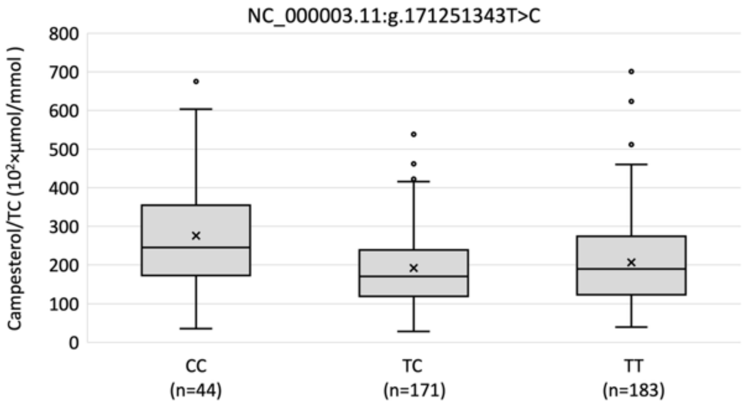

rs260769

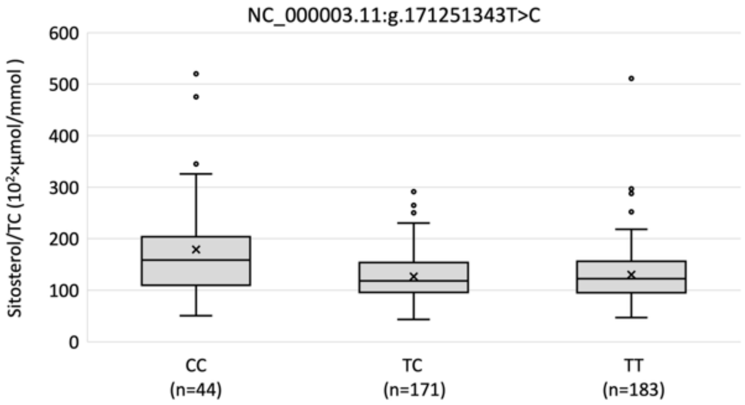

Figure S10. Boxplots for rs260769

rs5011112

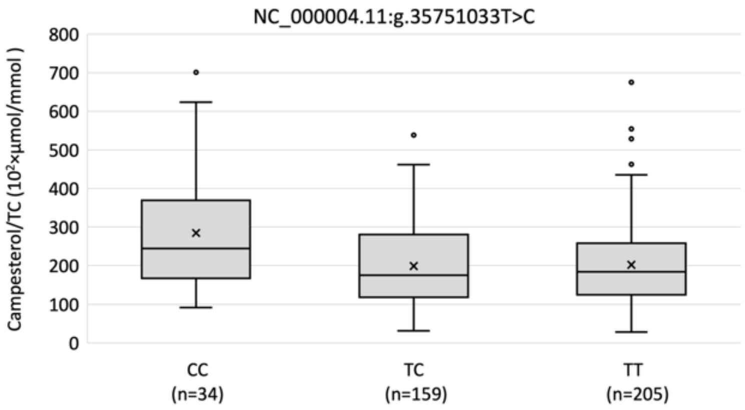

rs5011112

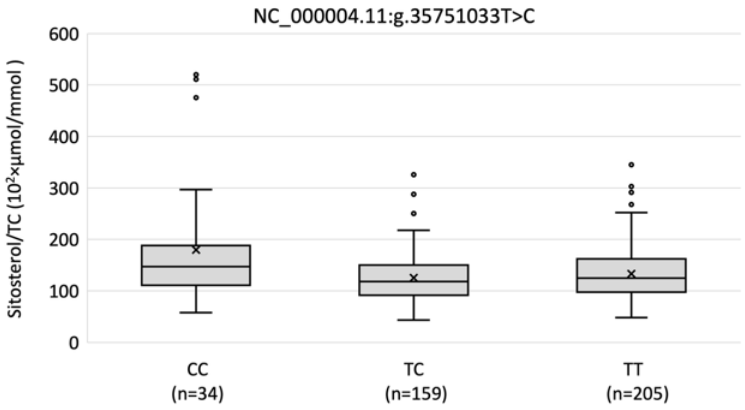

Figure S11. Boxplots for rs5011112

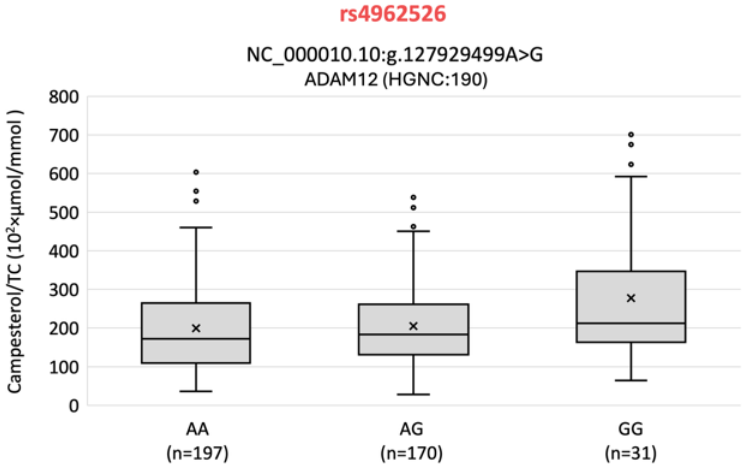

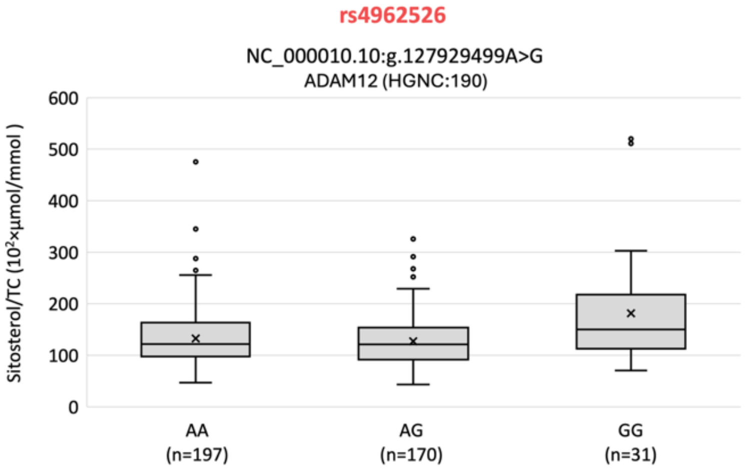

Figure S12. Boxplots for rs4962526

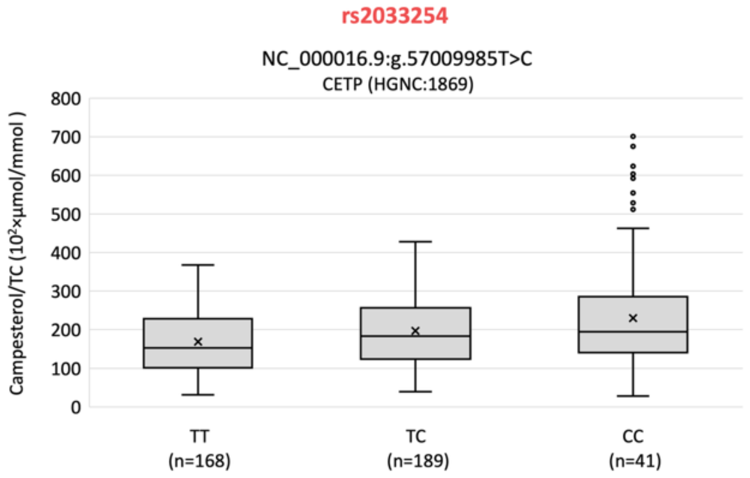

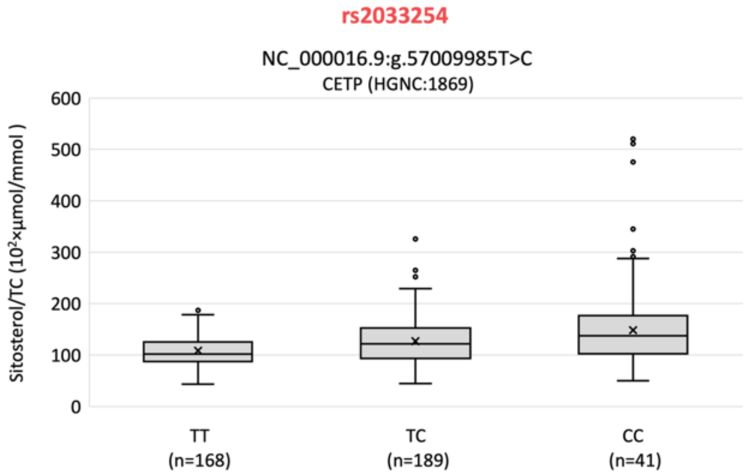

Figure S13. Boxplots for rs2033254

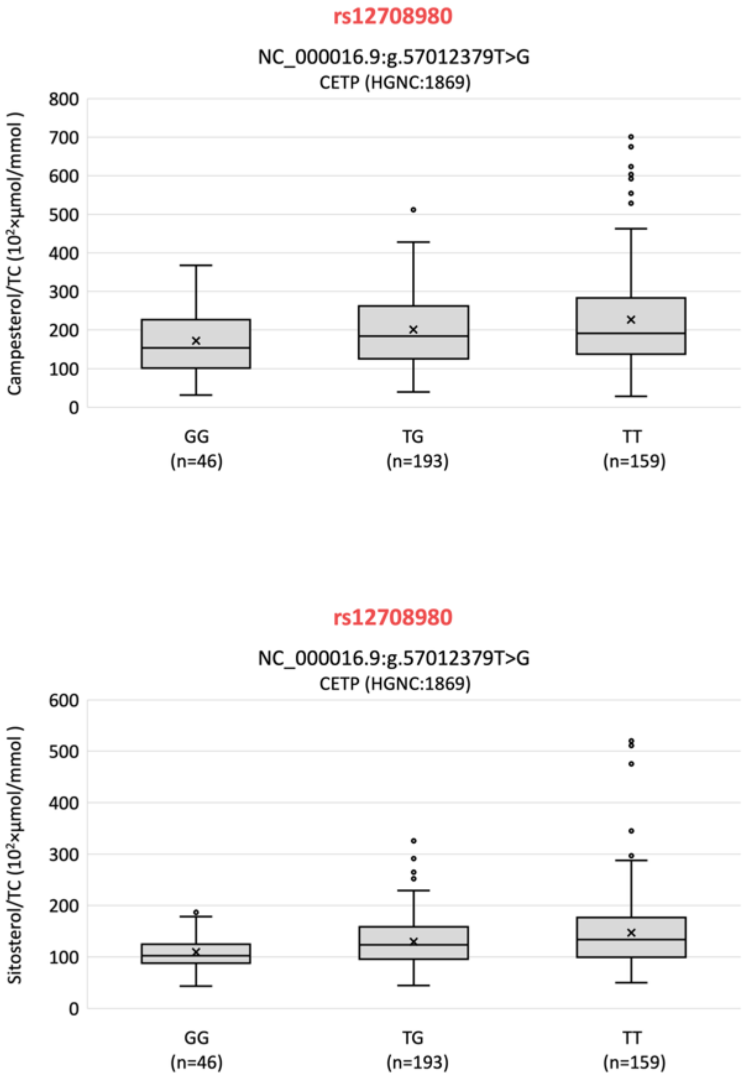

Figure S14. Boxplots for rs12708980
